# Arginine vasopressin: Direct and indirect action on metabolism

**DOI:** 10.1016/j.peptides.2021.170555

**Published:** 2021-08

**Authors:** Mitsuhiro Yoshimura, Becky Conway-Campbell, Yoichi Ueta

**Affiliations:** a Department of Physiology, School of Medicine, University of Occupational and Environmental Health, Japan; b Translational Health Sciences, Bristol Medical School, University of Bristol, UK

**Keywords:** Arginine vasopressin, Hypothalamus, Circadian rhythm, Energy balance, HPA axis

## Abstract

From its identification and isolation in 1954, arginine vasopressin (AVP) has attracted attention, not only for its peripheral functions such as vasoconstriction and reabsorption of water from kidney, but also for its central effects. As there is now considerable evidence that AVP plays a crucial role in feeding behavior and energy balance, it has become a promising therapeutic target for treating obesity or other obesity-related metabolic disorders. However, the underlying mechanisms for AVP regulation of these central processes still remain largely unknown. In this review, we will provide a brief overview of the current knowledge concerning how AVP controls energy balance and feeding behavior, focusing on physiological aspects including the relationship between AVP, circadian rhythmicity, and glucocorticoids.

## Highlights

• The roles of AVP on metabolism are described.
• A brief overview of the current knowledge concerning how AVP controls energy balance and feeding behavior is provided.
• We focused on physiological aspects including the relationship between AVP, circadian rhythmicity, and glucocorticoids.

## 1 Introduction

Arginine vasopressin (AVP), also known as antidiuretic hormone, is a hormone that is synthesized as a peptide prohormone, primarily in hypothalamic neurons. AVP is composed of 9 amino acids, Cys-Tyr-Phe-Gln-Asn-Cys-Pro-Arg-Gly−CONH_2_, cross-linked with a Cys-Cys disulfide bond in a ring structure. Historically, Resnik and Geiling first reported that pituitary extracts could affect the heart by stimulation of the vagal nerves through the cardio-inhibitory center and by direct action on the myocardium in 1925 [1]. In 1929 the word “vasopressin” first appeared in an article by David and Vareed [2], yet it wasn’t until 1954 that AVP was isolated and identified by du Vigneaud et al. [3]. During those decades, most of the AVP research focused on peripheral effects such as vasoconstriction, reabsorption of water from kidney, and regulating vagal nerve tone [4,5]. Since the cloning and characterization of three different types of AVP receptors in the 1980s [[6], [7], [8]], AVP has started attracting more attention as a “central” affecting neuropeptide as well as a peripheral peptide.

In a broad range of vertebrate and invertebrate species, the structure of AVP is highly conserved. For example, most vertebrate classes except mammals possess the nine amino acid peptide form, arginine vasotocin (AVT) which is a homologue of AVP. AVP differs only in position 3, with Ile being substituted for Phe [9]. AVT receptor as well is also highly similar to AVP receptor [10]. The structural conservations are paralleled with highly similar neural distribution of AVT and AVP, indicating evolutionary conservation in structure, expression patterns, and function for this ancient molecule.

AVP, which is produced in the hypothalamus, travels down axons terminating in the posterior pituitary, where it is released from vesicles into the systemic circulation in response to extracellular hyperosmolality. AVP is also released directly into the central nervous system (CNS) by somato-dendritic release [11]. Many ambitious studies have unveiled critical roles of centrally released AVP in various kinds of behaviors, including social recognition [12,13], pair bonding [14,15], aggression [15,16], and feeding behavior [[17], [18], [19]].

In addition, AVP also affects hypothalamic-pituitary-adrenal (HPA) axis. AVP released from terminals of parvocellular neurons of the paraventricular nucleus (PVN) stimulates adrenocorticotropic hormone (ACTH) synthesis and thus modulates glucocorticoid (GC) release from adrenal gland [20]. GC are known to have an orexigenic action by synaptic changes and altered excitability of the melanocortin system [21]. Furthermore, AVP produced in the suprachiasmatic nucleus (SCN) plays a crucial role in forming circadian rhythmicity [22,23], which may also be important for feeding regulation.

Taken together, although the AVP produced in each nucleus has different physiological roles, synchronization of AVP synthesis, transport, and release, both in the systemic circulation and in the CNS, may be essential for optimal feeding regulation and maintenance of energy balance. In this review, we aim to explore the basic roles of AVP, especially on feeding behavior and energy balance. We also provide integrated insights into AVP and the circadian rhythm, as well as our viewpoints about the relationship between AVP and GCs revealed in recent studies.

## 2 Distribution and receptors of AVP

In the mammalian brain, AVP is predominantly synthesized in the hypothalamus; magnocellular vasopressinergic neurons of the PVN, supraoptic nucleus (SON), parvocellular vasopressinergic neurons of the PVN, and accessory nuclei located between the SON and PVN [24]. In the PVN, AVP is produced in parvocellular neurons projecting to the median eminence, brainstem autonomic nuclei, and spinal cord [25,26]. In the SCN, AVP is produced in the shell, which is the dorsomedial part of the SCN [27]. AVP is also expressed in the medial amygdala, bed nucleus of the stria terminalis, diagonal band, dorsomedial hypothalamus, locus coeruleus, nucleus of the solitary tract and dorsal horn [28,29]. In addition, retinal cells in the eye have the ability to synthesize endogenous AVP, along with V1a and V1b receptor co-expression [30].

There are three types of known AVP receptors; V1a, V1b (also called V3), and V2, all of which are formed of heptahelical G protein-coupled receptors. Each receptor has relatively homologous amino acid sequences, however, their expression pattern is different among the tissues and organs. Regions of expression and their putative functions involved in metabolism are listed in the table (Table 1).

**Table 1:** Distribution of AVP receptors and their putative functions.

CNS	Expressed region	Putative function
V1a	Prefrontal cortex	Pair-bonding behavior
	Cingulate cortex	Social interaction
	Pyriform cortex	Social recognition
	Entorhinal cortex	Aggression
	Presubiculum	Maternal behavior
	Mamillary bodies	Anxiety-like behavior
	Amygdala	Depression
	Bed nucleus of the stria terminalis	Maintaining circadian rhythm
	Lateral septum	
	Hypothalamus	
	Brainstem	
V1b	Pituitary corticotropes	Development
	Olfactory bulb	Aggression
	Caudate putamen	Anxiety
	Septum	Thermoregulation
	Cerebral cortex	Alcohol preference
	Hippocampus	Chronological adaptation
	Hypothalamus	
	Cerebellum	
V2	AVP neurons in the hypothalamus	Volume regulation of AVP neurons
	Choroid plexus	
	Hippocampus	
	Granular layer of the Cerebellum	

Peripheral	Expressed region	Putative function
V1a	Lung	Vasoconstriction
	Liver	Lipolytic action
	Kidney	Enhance insulin sensitivity
	Smooth muscle cells	
	White adipose tissue	
	Brown adipose tissue	
V1b	Kidney	Anti-lipolytic action
	Thymus	Glycogenolysis
	Heart	Increase glucagon secretion
	Lung	Increase insulin secretion
	Spleen	Suppress insulin sensitivity
	Uterus	
	Breast	
	Liver	
	Pancreas	
	White adipose tissue	
V2	Renal distal tubules	Water reabsorption
	Renal collecting ducts	Insulin sensitivity
	Heart	
	Liver	
	Muscle	
	White adipose tissue	
	Brown adipose tissue	

V1a receptor is expressed in the vascular smooth muscle cells and CNS. V1a expressed in the vascular muscle cells is responsible for the vasoconstriction [31], whereas that expressed in the CNS influences a wide variety of brain functions, including pair-bonding behavior [15,32], social interaction and social recognition [33,34], aggression [35,36], maternal behavior [37], anxiety-like behavior [13], depression [38], and maintaining circadian rhythm [39]. The distribution of V1a receptor, investigated by *in situ* hybridization histochemistry, was consistent with a role for AVP in higher cognitive functions, including the prefrontal, cingulate, pyriform, and entorhinal cortex, as well as the presubiculum and mamillary bodies in rhesus monkeys [40]. V1a receptor binding and mRNA were also detected in the amygdala, bed nucleus of the stria terminalis, lateral septum, hypothalamus and the brainstem [40].

V1b receptor, which is expressed in the anterior pituitary, modulates the secretion of ACTH [41]. V1b receptor is also expressed in multiple brain regions, including pituitary corticotropes, olfactory bulb, caudate putamen, septum, cerebral cortex, hippocampus, hypothalamus, and cerebellum, as well as peripheral tissues, including kidney, thymus, heart, lung, spleen, uterus, and breast in rats [42,43].

V2 receptor is mainly expressed in the renal distal tubules and collecting ducts, and stimulates water reabsorption [44]. Sato et al., have reported that V2 receptor is also expressed in AVP neurons in the CNS [45]. V2 receptor in AVP neurons is considered to act as an autocrine signal, thus facilitates volume regulation of AVP neurons themselves. In the CNS, V2 receptor is expressed in the choroid plexus, and the neurons of the hippocampus and granular layer of the cerebellum, although the expression pattern was different depending on the age of the rats [46]. V2 receptors in the CNS may be associated with development [46], aggression [47], anxiety [48], thermoregulation [49], alcohol preference [50], and chronological adaptation [51].

In rats, a sex difference of AVP neurons and their dendrites was reported. Males had significantly more AVP-immunoreactive neurons in the bed nucleus of the stria terminalis and denser projections from this nucleus to the lateral septum, lateral habenular nucleus, and periaqueductal central gray than females [52]. Denser AVP-immunoreactive fiber networks were also found in the medial amygdaloid nucleus and ventral hippocampus, which receives its input from the medial amygdaloid nucleus [52]. Thus, in addition to AVP levels and the expression levels of its receptors, these sex differences should be considered when considering the function of AVP.

The signal transduction pathways of these receptors are characteristic. When the ligand, AVP, binds to V1a and V1b receptors, phospholipase C is activated via the Gq coupled protein, followed by upregulation of inositol 1, 4, 5- trisphosphate and diacylglycerol. Cellular or neuronal activities are thus regulated by virtue of the increase of intracellular calcium concentration and protein kinase C. On the other hand, in the V2 type receptor, adenylate cyclase is activated via Gs coupled protein [53]. Cellular or neuronal activities are thus regulated by cyclic AMP-dependent protein kinase.

Oxytocin, another peptide also produced in the magnocellular division of the SON and PVN, can also bind to AVP receptors with reduced affinity, and *vice versa* [54]. In addition, the ability of AVP to bind to the receptors could vary between the different organs, which may also modified by pathophysiological conditions. Thus, we should take into account which receptor plays a principal role for the specific behavior under each specific condition.

## 3 AVP and food intake

Recent views suggest that AVP reduces feeding in mammals. In the 1990s, Meyer and colleagues revealed that AVP injected intraperitoneally into pygmy goats reduced food intake in a dose dependent manner, specifically by reducing the size of the first meal and increasing the first inter-meal interval, [55]. Reduced food intake after intraperitoneally administered AVP was also observed in adult male rats [56]. These hypophagic phenomenon were, at least in part, mediated by α1-adrenergic receptors [56,57]. While hunger usually occurs in the absence of, or prior to, absorption of nutrients, only some of the signals that inhibit food intake can be associated with caloric homeostasis as food intake will be decreased after administration of nauseogenic chemical agents [[58], [59], [60]], or dehydration [61,62].

Dehydration-induced anorexia involves an important physiological adaptation that limits the intake of osmolytes from food and helps maintain the integrity of fluid compartments [62]. Watts and colleagues found that rats develop profound anorexia from dehydration when given hypertonic saline (2.5 % NaCl) instead of water [63]. They also showed that, in dehydrated rats in comparison with euhydrated rats, gene expression of *neuropeptide Y* (*NPY*) in the arcuate nucleus was significantly increased, *CRH* in the PVN was markedly decreased, and *CRH* in the lateral hypothalamic area was significantly increased [61]. Gene expression of *AVP* is also upregulated as a result of the hyperosmotic state. Indeed, 2% salt-drinking rats had increased *AVP* and *V1bR* mRNAs in the SON, PVN, and in the choroid plexus compared to rats maintained on water [64]. Taken together, these findings lead to the hypothesis that increased AVP could be involved in an anorexigenic effect in dehydration-induced anorexia.

The anorexigenic effect of AVP seems to be largely mediated through V1a receptor. Ikemura and colleagues demonstrated that food intake suppression in male rats after intraperitoneally administered AVP was attenuated by simultaneous injection of a peptide antagonist for V1 receptor, but not for V2 receptor [65]. Orexin, which is a critical peptide for sleep/wake cycles, is also involved in feeding behavior. AVP directly induced depolarization and an inward current in orexin neurons, while these were inhibited by a V1a receptor selective antagonist and were not observed in V1aR knockout mice [66]. NPY, an orexigenic peptide produced in the arcuate nucleus, potently increases food intake, and simultaneously stimulates arginine-vasopressin (AVP) secretion in the brain. The orexigenic effect of NPY was enhanced in V1a receptor knockout mice [67]. In WT mice, NPY-induced orexigenic effect was enhanced by intracerebroventricular administration of an antagonist for V1a receptor, but not for V1b receptor, an important role in blunting the orexigenic effect of NPY through a V1a mediated mechanism [67].

Similar results were obtained from the studies of avian AVT V1a receptor, which is highly homologous to mammalian AVP V1a receptor, and is associated with the regulation of food intake in chickens by modulating neurons that synthesize and release anorexigenic neuropeptides [68,69].

**Fig. 1:**
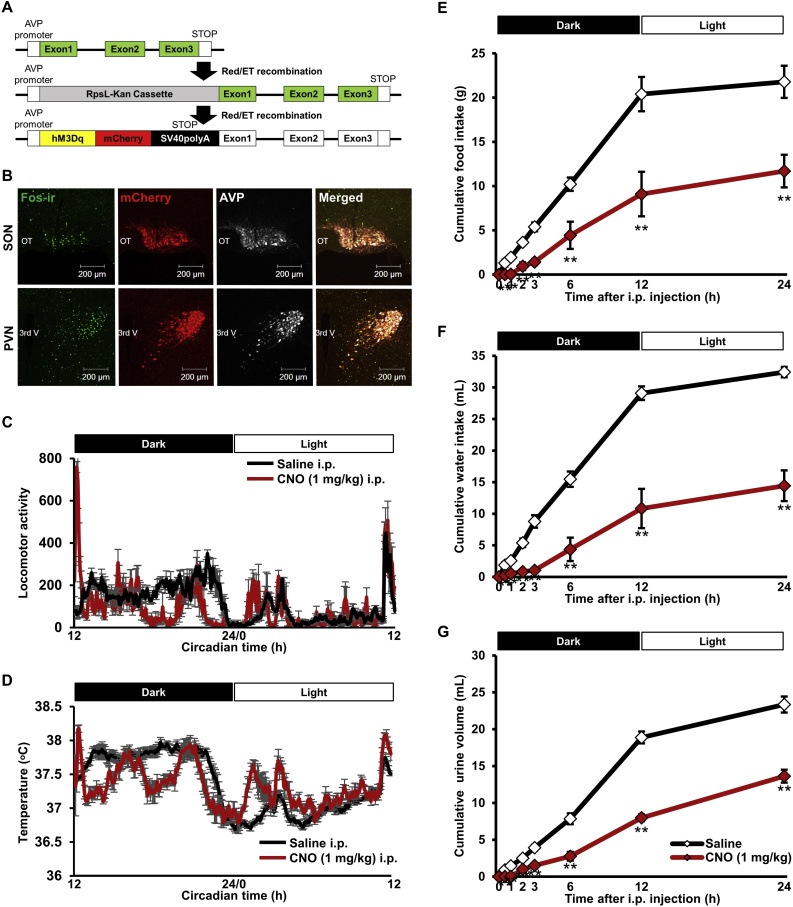
Chemogenetic activation of AVP neurons using transgenic rats. (A) Construction strategy of an AVP-hM3Dq-mCherry transgenic rat line are shown. (B) Robust Fos induction was observed 90 min after intraperitoneal (i.p.) injection of clozapine-N-oxide (CNO, 1 mg/kg) in the SON and PVN. Circadian activity (C) and circadian core body temperature (D) were significantly disrupted after chemogenetic activation of AVP neurons at the start of the dark phase. Cumulative food intake (E), water intake (F), and urine volume (G) were significantly suppressed after chemogenetic activation of AVP neurons. The figure was modified from Sci. Rep. 7, 2017 [17].

Recently, Pei and colleagues have demonstrated that activation of endogenous AVP in the PVN reduced food intake by using designer receptors exclusively activated by designer drugs (DREADDs) in mice [18]. We have gone on to show that activation of AVP expressing neurons decreased food intake and disturbed circadian rhythmicity of core body temperature and locomotor activity, by using a transgenic rat line that expresses excitatory DREADDs specifically in AVP neurons (Fig. 1) [17]. Decreased food intake observed in this study may be driven by AVP in the SON and PVN as well as by the SCN because AVP producing neurons in all these nuclei were activated. Although which of these nuclei was crucial for anorexigenic action of AVP remains unknown, we speculated that AVP produced in the SON and PVN may affect food intake directly, and AVP in the SCN may affect food intake indirectly by altering circadian rhythmicity. The results of these studies support an important role for AVP as an anorexigenic molecule.

In human, a strong association was observed between the RS3 microsatellite polymorphism in the V1a receptor promoter region and eating behavior [70]. The patients with RS3 microsatellite polymorphism in the V1a receptor promoter region appeared to attempt severe dietary restriction for weight loss purposes. The result suggested that aberrant *V1a receptor* expression is one of the potent factors that drives abnormal eating behavior.

In practical terms, not only AVP and/or its receptors, but also higher brain function is also important for forming eating behavior in humans. Given that higher brain function is much more advanced in humans compared to rodents, it is indeed difficult to explain the anorexigenic action of AVP in humans only from the aspect of the protein itself or from its receptors.

## 4 AVP and metabolism

AVP also modulates diverse metabolic functions, such as cellular growth and proliferation [[71], [72], [73]], protein turnover [74], lipid metabolism [75,76], and glucose homeostasis [77,78]. Variation of free fatty acid (FFA), glucose and insulin levels are monitored by metabolic sensing neurons located in the hypothalamus along with other gut hormones to alter their activity and act both on central and peripheral mechanisms that are involved in regulation of food intake. In this section, we focus on the function of AVP and AVP receptors that are important for regulating body energy balance.

### 4.1 AVP and lipid metabolism

AVP mediates lipid metabolism via a wide range of central and peripheral actions. AVP neurons in the PVN stimulate sympathetic nerve activity via axonal projections to the rostral ventrolateral medulla (RVLM), mediated via V1a receptor [79,80]; a mechanism with potential impact upon lipid metabolism in liver or adipose tissue. Although, specific mechanism of AVP should be further explored, there are some studies that reported sex difference regarding lipid and glucose metabolism. Indeed, lipoprotein profiles were altered in women after menopause, and this could be partially reversed by exogenous hormone replacement therapy [81] demonstrating an influence of gonadal hormones in regulating the lipoprotein profile.

AVP appears to have both lipolytic and anti-lipolytic actions depending on the experimental condition. For example, under starved state, constant infusion of AVP induced fatty acid release from adipose tissue by a direct anti-lipolytic effect in adipose tissue in rats [82], indicating that the primary metabolic effect of AVP in the starved state was due to decreased supply of non-esterified fatty acids to the liver *in vivo*. On the other hand, pitressin, a synthesized form of AVP, induced lipolysis in rat adipose tissue *in vitro* [75]. Küchler and colleagues showed that uncoupled protein-1 (UCP-1) expression was induced after acute exposure of AVP in differentiated brown adipocytes [83], suggesting that AVP has adipotropic effect, though the reason for these discrepant results remain unclear.

Actions of AVP on lipid metabolism are mediated through V1a and V1b receptors directly or indirectly. In adipose tissue, V1a receptor is expressed both in white adipose tissue (WAT) and brown adipose tissue (BAT), while V1b receptor is only expressed in WAT [84]. Hiroyama and colleagues demonstrated in an elegant way that serum carnitine and acylcarnitines were significantly increased and lipid metabolism was enhanced in response to isoproterenol by using V1a receptor-deficient mice [84]. These results indicate that beta-oxidation was promoted in these mice and that AVP could modulate the lipid metabolism by an anti-lipolytic action via the V1a receptor. They also demonstrated the function of V1b receptor on lipid metabolism by using V1b receptor-deficient mice [85]. The effect of V1b receptor on lipid metabolism seems to be opposite to that of V1a receptor. V1b receptor-deficient mice had, with exhibiting lower body weight, greater epididymal fat mass than wild type mice. Isoproterenol-stimulated lipolysis in differentiated adipocytes was significantly decreased in these mice with impaired insulin secretion and low blood glucose level. These results indicate that insulin sensitivity was increased as a compensatory mechanism, thus consequently anti-lipolytic effect could be induced in V1b receptor-deficient mice. From these studies, it is considered that lipid metabolism is altered by AVP, by modulating insulin signaling via V1a and V1b receptors. V1a receptor seems to be involved in exacerbating glucose tolerance and leading lipolytic action of AVP, while V1b in improving glucose tolerance and leading anti-lipolytic action of AVP [84,85]. However, glucose tolerance was impaired in V1a and V1b receptor double deficient mice [86]. These results indicate that AVP may exhibit anti-lipolytic effect rather than lipolytic effect *in vivo*.

In addition, AVP is involved in thermoregulation as one of the antipyretic hormones in peripheral tissue as well as in the CNS [[87], [88], [89]]. Since metabolic rate will be suppressed under hypothermic state [90,91], anti-lipolytic effects of AVP may be induced by hypothermia, an indirect action of AVP, as well as a direct action of AVP.

### 4.2 AVP and glucose metabolism in rodents

AVP appears to induce hyperglycemia, yet also improve glucose tolerance in animal experiments. Acute injection or sustained infusion of high dose of AVP induced a transient rise in blood glucose concentration in animals and humans [77,82,92]. These may be the results of an enhanced glycogenolysis induced by increasing glycogen phosphorylase via V1a receptor in the liver [77,93] and an increased glucagon secretion via V1b receptor from the alpha cells of the pancreas [[94], [95], [96]]. Glycogenolysis could also be stimulated by indirect action of AVP on vasoconstriction, followed by hypoxia in the liver [97]. In contrast, AVP increases insulin secretion via V1b receptor from the beta cells of the pancreas [95,98], though the dosage of AVP on its secretion is different from that in alpha cells [99]. The secretion of glucagon or insulin by administration of AVP depends on the conditions of glucose concentration; insulin secretion is highly induced by AVP under high glucose condition, while glucagon secretion is much increased by AVP under low glucose condition [99]. Since increased glucose concentration could stimulate intracellular concentration of calcium in beta cells in the pancreas, AVP can act as a positive modulator for glucose-stimulated insulin release [99,100]. AVP is reported to be expressed in the pancreas by RT-PCR [101], suggesting that not only circulating AVP but also possible paracrine function of AVP produced in the pancreas affects glucagon and/or insulin secretion.

A rise in blood glucose induced by an acute injection of AVP was prevented by pretreatment of V1a receptor antagonist, but was increased by treatment with V1a receptor agonist in 6 h fasted rats [102]. In these rats, V1b agonist nor antagonist did not change blood glucose level induced by acute injection of AVP, however, V1b receptor antagonist enhanced the fall in glucagon secretion after AVP injection [102].

AVP may enhance insulin sensitivity via the V1a receptor and suppress sensitivity via the V1b receptor. Considerable knowledge about the role of AVP in glucose homeostasis has been obtained from ambitious studies using animal models of V1a and/or V1b receptor-deficient mice. Impaired glucose tolerance was observed in mice lacking V1a receptor, without affecting plasma insulin levels [103]. Interestingly, overt obesity was induced by high-fat diet in V1a receptor-deficient mice compared to WT mice [103], which may indicate a possible involvement of V1a receptor on energy accumulation and/or expenditure as well as glucose homeostasis. Since AVP directly regulates aldosterone secretion through the V1a receptor [104], lower plasma aldosterone levels could contribute to a lower response to water retention from kidney. In V1b receptor-deficient mice, under fasted state, plasma insulin, glucagon, and blood glucose were decreased compared to wild type mice [105]. AVP effect on insulin release was ablated in pancreatic islets derived from V1b receptor deficient mice with lower plasma glucose level [106], suggesting that insulin hypersensitivity is present under V1b receptor deficient condition. In addition, since plasma ACTH was decreased in V1b receptor-deficient mice in comparison with wild type mice [41], HPA axis modulation could also be involved in altered glucose homeostasis in these mice. The glucose homeostasis phenotype of V1a and V1b receptor-double-deficient mice is similar to that of V1a receptor deficient mice [107].

Brattleboro rats, which genetically lack AVP, are used to delineate the global action of AVP on glucose homeostasis. These rats showed enhanced glucose tolerance instead of impaired glucose tolerance [107]. The discrepancy of these results obtained from V1a and V1b receptor-double-deficient mice and Brattleboro rats may indicate that there is possible involvement of V2 receptor as well as V1a and V1b receptors in regulating glucose homeostasis *in vivo*. V2 receptor is expressed in many different insulin sensitive tissues, including heart, liver, muscle, WAT, and BAT [105]. Together, these may support the hypothesis that V2 receptor signaling may also, at least partially, be involved in glucose homeostasis by altering not only the regulation of water balance but also insulin signaling.

Taveau and colleague have demonstrated that, in Zucker fatty rats, which genetically have a mutation in the leptin receptor gene, fasting hyperglycemia as well as hyperinsulinemia was induced after chronic intraperitoneally injection of AVP for 4 weeks [108]. Hyperglycemia induced by chronic AVP infusion was diminished by concomitant treatment with V1a receptor antagonist, whereas, insulin levels were the same as the group that had normal AVP concentration. Interestingly, in their study, low AVP induced by high water intake did not improve glucose tolerance in Zucker rats, although they did have a lower incidence of liver steatosis. Despite the lack of a detailed elucidation of the underlying mechanism, these findings have demonstrated that there might be a causal relationship between the AVP-hydration axis and metabolic adverse effects.

Based on the results from animal experiments, although AVP induces hyperglycemia, it also reduces food intake and improves glucose tolerance. It therefore appears that together these result in balancing glucose metabolism.

### 4.3 AVP and glucose metabolism-related disease in human

While AVP appeared to improve glucose tolerance in animal experiments, recent findings have revealed an independent association between plasma copeptin, which is a stable C-terminal portion of pre/pro-vasopressin peptide and is used for a surrogate marker for circulating AVP, and risk of diabetes. AVP levels were indirectly measured in some studies, perhaps explaining the discrepancy, and raising a question about whether copeptin is an appropriate surrogate for AVP in diabetes.

In human, the mean basal plasma AVP level in the patients with diabetes mellitus was significantly higher than control subjects [109]. Many clinical studies have suggested that high blood AVP levels, or high blood copeptin levels, could contribute to type 2 diabetes mellitus and metabolic syndromes [[110], [111], [112], [113]]. In addition to its correlation with type 2 diabetes mellitus and metabolic syndrome, higher blood copeptin levels are also associated with high fat intake, lower physical activity and lower socio-economic status. Plasma copeptin is also associated with the presence and severity of nonalcoholic fatty liver disease and steatohepatitis (NAFLD/NASH) [114,115]. Another study has shown that the amount of daily water intake was negatively associated with the risk of developing hyperglycemia or type 2 diabetes mellitus in a 9-year follow-up study [116]. Baseline plasma copeptin was also positively and independently associated with the later incidence of microalbuminuria, abdominal obesity, and hypertension in a 15.8-year follow-up study [117]. Enhörning and colleagues have suggested that, specifically in the patients with diabetes, copeptin could predict heart disease and death therefore it could be potential target for diabetic heart disease and death [118]. Plasma copeptin level is higher not only in patients with type 2 diabetes mellitus but also in patients with type 1 diabetes mellitus [119]. A recent study has revealed that copeptin did not correlate with markers of insulin resistance in type 1 diabetes mellitus but strongly correlates in non-type 1 diabetes mellitus [120]. Plasma copeptin levels were lower in individuals with bipolar disorders in comparison to healthy controls. Interestingly, there were significant interactions between plasma copeptin on β-cell function and plasma leptin levels only in the subjects with bipolar disorders but not in healthy controls [121]. Canivell and colleagues have shown that age and apparent 11β-hydroxysteroid dehydrogenase type 2 (11β-HSD2) activity modulate the association of copeptin with insulin resistance but not metabolic syndrome nor type 2 diabetes mellitus [122].

Plasma basal copeptin is higher in males in comparison to females, though the clinical significance has not been clarified yet. Stronger association has been reported in women than in men between baseline plasma copeptin and the incidence of type 2 diabetes mellitus [123]. On the contrary, Then and colleagues have reported that plasma copeptin was associated with type 2 diabetes mellitus in men but not in women [124]. Copeptin was also significantly associated with an increased risk of type 2 diabetes mellitus in older men, which was partly mediated through lower insulin sensitivity [125]. Dabrowski and colleagues have studied if plasma copeptin could be a useful biochemical marker of insulin resistance in pregnant women with early and late manifestation of gestational diabetes mellitus. According to their study, serum copeptin concentration was not useful to discriminate between early and late onset of gestational diabetes mellitus [126].

As well as the effects on lipid metabolism, it is speculated that AVP may play a differential role on glucose metabolism between males and females. Interestingly, males are more likely to develop elevated fasting glucose levels, whereas females are more likely to develop impaired glucose tolerance [127]. These may be caused by effects of gonadal hormone-dependent and -independent sex differences in regional adipose tissue distribution, production of cytokines and adipokines, hepatic gluconeogenesis and glycogenolysis, and glucose uptake by skeletal muscle [128].

Genetic epidemiology of AVP and its receptors in metabolic disorders has also been investigated in human. For example, significant associations were observed between the tagSNPs of the AVP gene (CC genotype of rs6084264, the TT genotype of rs2282018, the C-allele of rs2770381, and the CC genotype of rs1410713) and the incidence of hyperglycemia and decreased insulin sensitivity [129]. T-allele of rs1042615, which is a tagSNP in the AVP receptor V1a gene, was associated with an increased prevalence of type 2 diabetes mellitus in subjects with a high fat intake or who are overweight [130]. A major A-allele of rs35810727, a tagSNP in the AVP receptor V1b gene, was associated with elevated body mass index (BMI), waist, and type 2 diabetes mellitus [131]. Thus, genetic variance of AVP and its receptors might contribute, at least in part, to develop metabolic disorders, including obesity, overweight, and type 2 diabetes mellitus.

According to these studies, modifying the AVP system could be a potential therapeutic target for glucose metabolism-associated disease in patients.

## 5 AVP and circadian rhythm

The SCN of the hypothalamus contains the master circadian pacemaker, which is mainly synchronized by light. AVP is produced in the SCN shell, which is the dorsomedial part of the SCN [27]. Besides AVP-producing neurons in the shell, there are vasoactive intestinal peptide (VIP)-producing neurons and gastrin releasing peptide (GRP)-producing neurons in the core, the ventrolateral part of the SCN. The afferents from the hypothalamus and limbic system terminate mainly in the SCN shell [132].

Daily expression pattern of heteronuclear (hn) AVP, which is an indicative of gene transcription, peaked at zeitgeber time (ZT)1 and ZT5, then decreased to undetectable levels at ZT17, while *AVP* mRNA peaked at ZT5 and ZT9 in the SCN [133]. Daily expression pattern of these mRNA is very similar to that of period 1 (Per1) gene [133]. AVP neurons in the retinal ganglion cells were also activated by light exposure [134]. These indicate that the gene expression of AVP in the SCN was up-regulated by light exposure.

It is important to discuss the effects of AVP on circadian rhythmicity, as circadian activity could be one of the major factors that impacts feeding timing, amount, and/or metabolism. AVP concentration in the cerebrospinal fluid (CSF), but not in the blood, reaches at a peak in the morning [[135], [136], [137]]. For example, with regard to body fluid homeostasis, Gizowski and colleagues have demonstrated that, using optogenetic technique in mice, anticipatory thirst was driven by excitatory peptidergic neurotransmission mediated by AVP release from the SCN [138]. We have previously shown, in a transgenic rat line, that chemogenetic activation of AVP neurons at the start of the dark period induced aberrant behavior, and affected core body temperature and food intake [17], whereas no effect was found when stimulating these neurons in the light phase. In human, light exposure early in the dark period shifted the circadian rhythm backward, whereas light exposure late in the dark period shifted the circadian rhythm forward [139]. Indeed, light exposure acutely suppressed food intake through SCN AVP neurons via SCN AVP - PVN oxytocin pathway [140]. Thus, it is speculated that light exposure does stimulate AVP neurons in the SCN in addition to synchronizing SCN neurons.

Circadian phase-shift (locomotor activity, body temperature, and *clock gene* expression) was immediately re-entrained in V1a and V1b receptor double knockout mice [51]. This indicates that an animal model which loses AVP-mediated inter-neuronal communication appears to be resistant to light/dark environmental perturbation, such as jet lag. In other words, inter-neuronal communication, which is mediated by AVP, is crucial for maintaining normal circadian rhythm. They also demonstrated that, in wild-type mice, pharmacological blockade of V1a and V1b in the SCN resulted in accelerated recovery from circadian phase-shift. This suggests the AVP signaling may be a potent therapeutic target for management of circadian rhythm misalignment, which would also manage metabolic disorders.

Because ubiquitous AVP knockout in mice is fatal, development of SCN-specific AVP knockout mice would be useful for elucidating further mechanism of AVP in the SCN.

Interestingly, some characteristics of the circadian system and body weight regulation differ between males and females in human. For example, circadian misalignment disrupts energy balance in females and males through different pathways; females had more disturbances in the energy homeostasis process, including a decrease in the satiety hormone and an increase in hunger hormone, while males had elevated cravings for energy-dense and savory foods [141]. Base on the results from the animal experiments, it is tempting to speculate that these differences between the male and female response to circadian misalignment may be partly due to sexually dimorphic neuroanatomical distribution of AVP neurons and their dendrites.

## 6 AVP and the HPA axis

When an animal encounters a stressful situation, either real or perceived, a rapid activation of the HPA axis ensues [142,143]. Such stressors span a diverse range, from cognitive stress (for example, an unexpected loud noise or a short period of inescapable restraint) to physiological stress (for example hypoglycemia) to immunological stress (for example infection). The key site that integrates the neuroendocrine response to stress is the hypothalamic paraventricular nucleus (PVN), which is comprised of two regions termed the magnocellular (mPVN) and parvocellular (pPVN) subdivisions. While the mPVN and supraoptic nucleus (SON) comprise the neurohypophysial system, which is the major source of AVP and Oxytocin released into the circulation from neurons terminating in the posterior pituitary, the pPVN is the main source of the primary HPA effector molecule CRH.

**Fig. 2:**
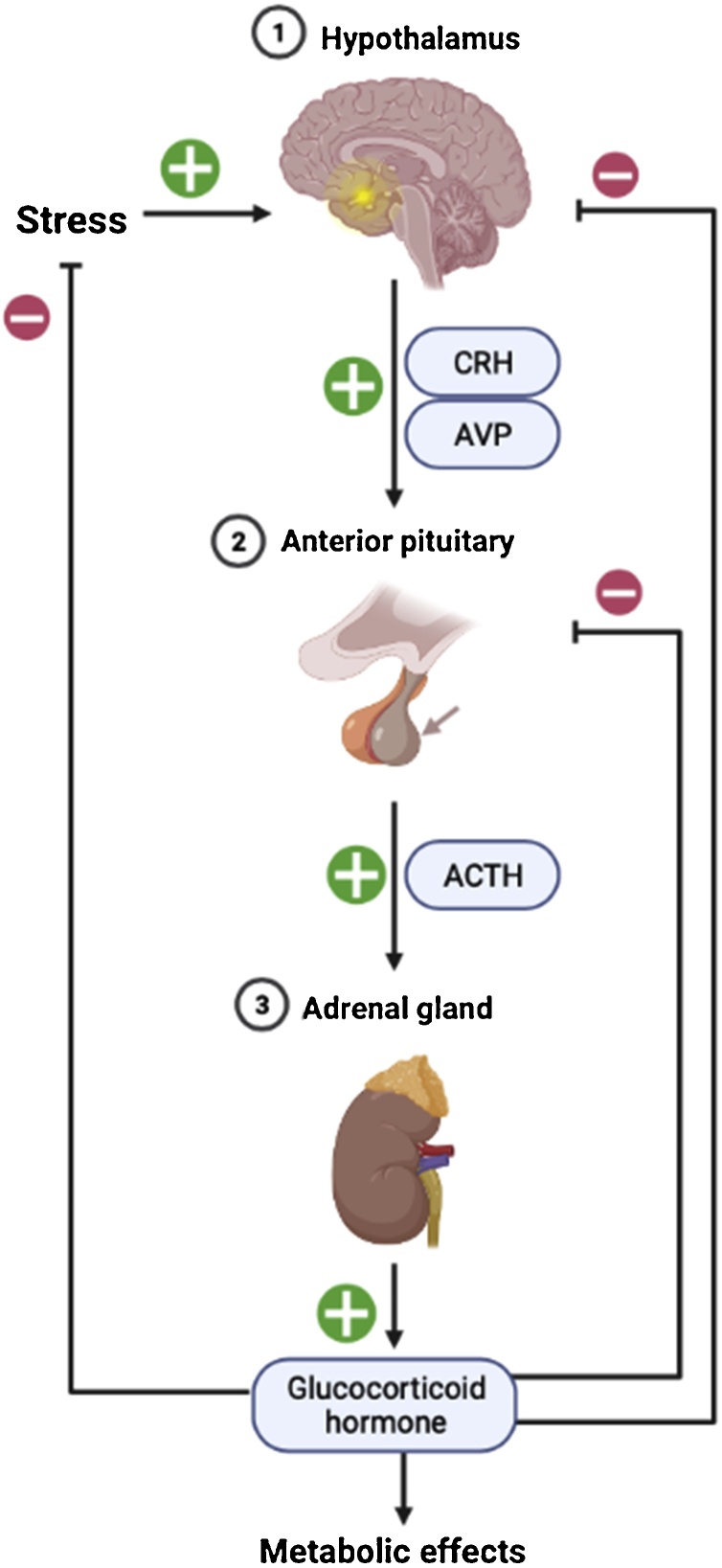
The HPA axis. In response to stress, CRH neurons originating from the pPVN and projecting to the median eminence, release both CRH and AVP into the portal circulation reaching the anterior pituitary. Here, CRH and AVP bind and activate the CRH-type1 receptor (Crhr1) and the V1b receptor respectively; the two cognate receptors that are expressed on corticotrophes within the anterior pituitary and stimulate ACTH secretion. ACTH travels through the peripheral ACTH circulation to reach the adrenal cortex where it induces steroidogenesis, effectively increasing secretion of glucocorticoid hormone (corticosterone in rodents and cortisol in humans). In turn, glucocorticoids enter the circulation and travel back to the pituitary and hypothalamus to act in a classical negative feedback loop to prevent further glucocorticoid release, effectively terminating the stress response Created with BioRender.com.

It is however important to note that approximately 50 % of pPVN CRH neurons also express AVP [144]; and that a stress such as acute restraint not only increases the levels of *CRH* but also increases the levels of *AVP* mRNA in these neurons [145]. Such restraint stress has been further shown to increase AVP synthesis in the pPVN, its accumulation in the median eminence, and its release into the hypophyseal portal system [145,146]. In contrast, *AVP* mRNA expression in the mPVN and the SON remains unaffected by the same stressor [147], highlighting region-specific differences in transcriptional control. It should also be noted that negative feedback mechanisms are not exclusively associated with transcriptional inhibition, but also directed at CRH/AVP peptide production and/or secretion. Fig. 2 shows an overview schematic of the HPA axis response to stress, with the feedforward arm of the response mediated by CRH and AVP. Negative feedback is subsequently directed at both CRH and AVP in pPVN neurons [146], and there is even some evidence for preferential inhibition of AVP compared with CRH [24,147,148]. GC inhibition is thought to act at multiple sites, inhibiting AVP secretion by direct actions on pPVN neurons, and indirectly by modifying the activity of neural pathways, with GABAergic and glutamatergic afferents to the parvocellular neurons [149,150]. GCs can also induce the release of endogenous cannabinoids, which can mediate rapid indirect, non-genomic GC feedback in parvocellular neurons [151].

Direct transcriptional inhibition of AVP by GCs has been shown to depend upon an active GC response element (GRE) within the AVP gene promoter [148], and further found to be independent of synaptic transmission in studies conducted in hypothalamic organotypic cultures [149], leading to the conclusion of direct inhibition of *AVP* gene transcription by GCs. Therefore, while it is widely accepted that non-genomic GCs actions are likely to mediate most rapid feedback effects [150], the direct inhibitory effects on CRH and AVP synthesis more likely account for long term feedback inhibition.

Although CRH is recognized as the primary ACTH secretagogue, it has been demonstrated that AVP can play a compensatory role in maintaining HPA activity in the absence of CRH. In elegant experiments using CRH and Crhr1 knockout mouse models, the intact vasopressin system was found to be sufficient to maintain adequate HPA activity for survival, although only after lung maturation has been completed with exogenous GC treatment [[151], [152], [153]]. Furthermore, consistent with the proposed compensatory role of AVP, a selective GC-dependent increase in the hypothalamic vasopressin system was found in the Crhr1 knockout mice [154].

There is also considerable evidence that AVP acts synergistically with CRH to enhance ACTH secretion [155]. AVP deficient Brattleboro rats have a blunted corticosterone response to some but not all stressors [156,157], while elegant studies using immuno-neutralization of AVP also reported a blunted HPA response to a range of stressors including restraint, insulin-induced hypoglycemia and lipopolysaccharide [[158], [159], [160]]. Studies, where pituitary portal blood was sampled, have been able to show that AVP is released preferentially over CRH in some cases, including insulin-induced hypoglycemia [[161], [162], [163], [164], [165], [166], [167], [168], [169], [170]].

There is strong evidence that AVP may become the dominant ACTH secretagogue in some chronic stress situations [171,172]. In particular, when the same type of stressful stimuli is repeated over a number of days, HPA axis desensitization leads to diminished stress responsiveness as an adaptative mechanism. This adaptive response appears to be largely dependent upon AVP and the corticotroph-expressed V1b receptor. For example, repeated restraint stress in rats continues to induce elevated AVP but not CRH expression in pPVN CRH-containing neurons [173]. Acute restraint following repeated restraint results in a rapid increase in AVP- but not CRH-hnRNA in the pPVN [174]. Finally, chronically restrained rats are able to respond to exogenous AVP treatment with increased ACTH levels, while exogenous CRH treatment has no effect on their ACTH levels [175]. In pituitary corticotrophs, both Crhr1 and V1b receptor are activated and undergo stress induced regulatory variations, but only the changes in V1b receptor levels are regulated in a manner that mirrors pituitary ACTH responsiveness [41].

Information gleaned from V1b receptor knockout mice highlight specific differences found with different types of stress exposure. One line of V1b receptor knockout mice exhibited significantly reduced ACTH and adrenal GC responses to the forced swim stress test [176] whereas another line of V1b receptor knockout mice exhibited normal adrenal GC response to the acute physical-psychological stress induced by the resident-intruder stress test [41] but significantly reduced ACTH response to a chronic homotypic stress paradigm of 14 daily inescapable restraint sessions [177]. Taken together, these groups’ data indicate that V1b receptor may be required for the normal pituitary and adrenal response to certain acute stressors, but only appears to play a role in maintaining pituitary corticotroph responsiveness during chronic stress. However, as potentially confounding compensatory mechanisms may sometimes arise in knockout mouse models, it will be important to assess the effects of modulating the AVP system with finer temporal control before we can fully understand the relative contribution of CRH and AVP in chronic stress, and particularly the consequent impact that any change in circulating GCs will have on metabolism and energy balance.

## 7 GC regulation of metabolism and feeding behavior

It has long been known that stress and GCs regulate metabolic [178,179] immunological [180] and cognitive processes [181,182] throughout the body and brain. Of particular relevance to the subject of this review, are the vast number of stress and GC-dependent effects on metabolism and energy balance, reviewed in [183,184]. Notably, the GC hormone is in its active form [185] and can regulate vast metabolic transcriptional networks [186] throughout metabolically active organs, including liver, adipose, skeletal muscle and pancreas [178]. The cognate glucocorticoid receptor (GR) is widely expressed throughout these tissues [187], therefore any AVP-dependent modulation of circulating GC levels as outlined above has the potential for a significant albeit indirect impact on metabolism and energy balance. For example, as one of the primary GC effects is to supply glucose to the body in conditions of acute stress or reduced food intake [187], GCs increase hepatic glucose production [178,188], decrease peripheral glucose uptake into muscle and adipose tissue [189,190] and increase breakdown of fat and muscle to provide additional substrates for glucose production [187,191,192]; as well as inhibit insulin release from pancreatic β-cells [193].

GCs also have effects on feeding behaviour. In contrast to AVP’s anorexigenic actions, GCs are orexigenic. In an elegant study using human volunteers, high cortisol secretion in response to an acute laboratory stressor was also related to voluntary increase in eating sweet, high-fat food after the stressor [194]. These types of associations between stress-induced GCs and increased feeding behaviour have been appreciated for decades, but elucidation of the mechanism whereby feeding rats highly palatable foods such as lard and sugar mediates downregulation of CRH in the amygdala, part of the neural stress circuitry [195,196] helps to explain the role of comfort food in stress coping behavior via HPA axis negative feedback mechanisms.

Therefore, it is important to understand how AVP can influence GC secretion as well as how the subsequent interplay between the two contribute to regulation of metabolism and energy balance.

## 8 Perspectives and conclusion

A role of AVP in metabolism has been elucidated by many ambitious studies. AVP itself has been demonstrated to exert direct actions on glucose and lipid metabolism. However, it is further speculated that indirect actions of AVP - for example via hemodynamic effects on adipose tissue, and via modulation of circadian rhythms and the HPA axis - will also exert significant influence on metabolic regulation.

In today’s modern society, sometimes described as a “nightless castle”, human health is being negatively impacted. We are exposed to light at all times of day and night; we are exposed to many stressors both physical and psychological.

Focusing on circadian rhythmicity, short term disruption causes jet lag-like symptoms whereas longer term disruption can lead to diverse metabolic disorders, including obesity, metabolic syndrome, type 2 diabetes, and cardiovascular diseases. Cancer risk, depression, and cognitive impairment are also increased by chronic circadian disruption [182]. A large number in the population are affected by chronic circadian disturbance, including day/night time rotation shift workers, pilots and flight crews, as well as patients with chronic sleep disturbance. Focusing on stress, the adaptive acute stress response becomes maladaptive when the stressful situation becomes chronic [197]. Increasing tendencies of overeating highly palatable ‘comfort food’ for coping with chronic stress [[194], [195], [196],^198^] is exacerbated by the metabolic effects of chronically elevated GCs [178], and most likely a contributing factor in the WHO 2021 World Health Organization’s reported current obesity epidemic, with the associated increase in incidence of metabolic syndrome and Type 2 Diabetes.

As AVP is intrinsically associated with both circadian rhythm and GC regulation, as well as exerting direct effects on feeding behavior and metabolic regulation in its own right, further elucidation of the underlying mechanisms of AVP control will contribute to the identification of potential therapeutic targets for the treatment of metabolic disorders.

## Declaration of Competing Interest

The authors report no declarations of interest.
